# Arginine Methyltransferase PeRmtC Regulates Development and Pathogenicity of *Penicillium expansum* via Mediating Key Genes in Conidiation and Secondary Metabolism

**DOI:** 10.3390/jof7100807

**Published:** 2021-09-27

**Authors:** Xiaodi Xu, Yong Chen, Boqiang Li, Shiping Tian

**Affiliations:** 1 Key Laboratory of Plant Resources, Institute of Botany, The Innovative Academy of Seed Design, Chinese Academy of Sciences, Beijing 100093, China; xuxiaodi@ibcas.ac.cn (X.X.); chenyong@ibcas.ac.cn (Y.C.); bqli@ibcas.ac.cn (B.L.); 2 College of Life Sciences, University of Chinese Academy of Sciences, Beijing 100049, China

**Keywords:** *Penicillium expansum*, protein arginine methyltransferase, development, pathogenicity, metabolism regulation

## Abstract

*Penicillium expansum* is one of the most common and destructive post-harvest fungal pathogens that can cause blue mold rot and produce mycotoxins in fruit, leading to significant post-harvest loss and food safety concerns. Arginine methylation by protein arginine methyltransferases (PRMTs) modulates various cellular processes in many eukaryotes. However, the functions of PRMTs are largely unknown in post-harvest fungal pathogens. To explore their roles in *P. expansum*, we identified four PRMTs (PeRmtA, PeRmtB, PeRmtC, and PeRmt2). The single deletion of *PeRmtA*, *PeRmtB*, or *PeRmt2* had minor or no impact on the *P. expansum* phenotype while deletion of *PeRmtC* resulted in decreased conidiation, delayed conidial germination, impaired pathogenicity and pigment biosynthesis, and altered tolerance to environmental stresses. Further research showed that PeRmtC could regulate two core regulatory genes, *PeBrlA* and *PeAbaA*, in conidiation, a series of backbone genes in secondary metabolism, and affect the symmetric ω-*N*^G^, *N’*^G^-dimethylarginine (sDMA) modification of proteins with molecular weights of primarily 16–17 kDa. Collectively, this work functionally characterized four PRMTs in *P. expansum* and showed the important roles of PeRmtC in the development, pathogenicity, and secondary metabolism of *P. expansum*.

## 1. Introduction

*Penicilliumexpansum* is a necrotrophic filamentous ascomycete and one of the most aggressive *Penicillium* species [1,2]. It causes blue mold rot in pome, stone and other stored fruit worldwide, bringing huge annual economic losses to the fruit industry [1,3]. *P. expansum* produces patulin, a potential carcinogenic mycotoxin that contaminates infected fruit and their products, giving rise to food safety concerns [4,5]. Sustained research efforts are focused on the extensive molecular mechanisms of the development and pathogenesis of *P. expansum* to provide a theoretical basis for control strategies. Several regulatory and virulence factors have been identified [3], but few studies refer to post-translational modifications and protein-modifying enzymes that functionally diversify the proteome and are involved in the regulation of gene expression [6,7]. 

Arginine methylation, an important post-translational modification, is catalyzed by protein arginine methyltransferases (PRMTs) and regulates cellular processes in a variety of living things, from yeast to mammals [8,9]. The PRMT family of enzymes is evolutionarily conserved and generally divided into four types [10]. Type I, II, and III enzymes catalyze ω-*N*^G^-monomethylarginine (MMA) while type I and II enzymes can further generate asymmetric ω-*N*^G^, *N*^G^-dimethylarginine (aDMA) and symmetric ω-*N*^G^, *N’*^G^-dimethylarginine (sDMA), respectively [11,12]. Type IV enzymes are responsible for δ-*N*^G^-monomethylarginine [13,14]. 

As reported, filamentous fungi harbor four PRMTs, which are homologous to mammalian PRMT1 (type I), PRMT3 (type I), PRMT5 (type II), and yeast RMT2 (type IV), respectively [15]. Homologs of PRMT1, PRMT3, and PRMT5 were found to affect the growth, development, secondary metabolism, and stress response of fungi to different extents, and PRMT1 homologs were involved in the virulence of a few important plant fungal pathogens, including *Aspergillus flavus*, *Fusarium graminearum*, and *Magnaporthe oryzae* [16,17,18,19,20]. In addition, there may be reciprocal regulation among homologs of PRMT1, PRMT3, and PRMT5 that affects transcriptional levels of *PRMTs* in *F. graminearum*, the arginine methylation profiles in *Neurospora* *crassa*, and the protein secretion, secondary metabolism, and detoxification in *A. nidulans* [15,21,22], whereas, the biological function of RMT2 homologs in fungal pathogens is not yet clear. 

This study aimed to identify PRMT homologs and explore their roles in *P. expansum*. We confirmed the importance of PeRmtC (type II PRMT) in development, pathogenicity, and secondary metabolism through a phenotype analysis of PRMTs deletion mutants. We further showed the action mode of PeRmtC was to regulate the expression of key genes in conidiation and secondary metabolism, and verified the impact of PeRmtC on the sDMA modification of proteins. 

## 2. Materials and Methods

### 2.1. Fungal Strains, Media and Culture Conditions

The wild type (WT) strain *P. expansum* T01 was isolated from infected apple fruit and whole-genome sequenced [23]. All strains in this study were stored at −80 °C and grown on potato dextrose agar (PDA) at 25 °C. Conidia from PDA plates were harvested through sterile gauze and counted using an automated cell counter (Countstar, IY1200, Shanghai, China).

### 2.2. Conserved Motif and Phylogenetic Analysis

The PRMTs homologous sequences in different fungal species were obtained by using the amino acid sequences of PRMTs in yeast as bait for BLASTp searches on NCBI (http://www.ncbi.nlm.nih.gov/, accessed on 14 February 2019). The sequences were aligned using BioEdit 7 and Clustal W. The graphic representation of the alignment and conserved motif was produced with ESPript (https://espript.ibcp.fr/ESPript/cgi-bin/ESPript.cgi, accessed on 12 July 2021). Phylogenetic analysis was performed using MEGA 6 software, and the maximum likelihood (ML) tree was generated with a bootstrap value of 1000 [24].

### 2.3. Construction of Gene Deletion and Complementation Strains 

Gene deletion and complementation strains in this study were generated in reference to the method described previously [25]. The hygromycin phosphotransferase gene *hph* was used as the resistance marker for the single-deletion construct, while the neomycin resistance gene *neo* was used for double-deletion and complementation constructs. Two homologous recombination sequences (5′ flank and 3′ flank) flanking the target gene were amplified from the WT genomic DNA and inserted at the multiple cloning sites on pCHPH (or pCNEO) upstream and downstream of *hph* (or *neo*), respectively. The positive transformants were purified by single spore isolation and confirmed by Southern blot analysis and PCR. For Southern blot analysis, 3-day-old mycelia were collected for genomic DNA extraction. The genomic DNA of ∆*PeRmtC* and ∆*PeRmt2* was digested with *Hind* III, and that of ∆*PeRmtA* and ∆*PeRmtB* with *EcoR* I and *Xbal* I, respectively. The digested genomic DNA was hybridized with an *hph*-specific probe that was amplified from the pCHPH vector and labeled by DIG. The primers are listed in Table S1. 

### 2.4. Phenotype Assay 

Growth rate and conidiation were detected as previously described [25]. An aliquot of 5 μL 1 × 10^5^ conidia mL^−1^ suspension was incubated on PDA plates for 10 d, and the colony diameter was measured after 3 d of incubation. Conidia were harvested after 3, 7, and 10 d for quantity determination. Three plates were used for each strain and the experiments were repeated in triplicate.

For the assay of conidial germination and germ tube elongation, 3 × 10^7^ conidia mL^−1^ suspensions were coated on cellophane sheets and cultured on PDA plates. The germination rate and germ tube length were determined by microscopic observation. Approximately 200 spores were randomly observed in each strain and the experiments were repeated three times. 

The moist chamber slide culture, modified from the method described previously [26], was performed to examine conidiogenesis. An aliquot of 3 μL 1 × 10^5^ conidia mL^−1^ suspension was incubated on the aseptic slide and allowed to dry, after which the incubation site was covered by a piece of PDA (5 × 5 × 1 mm) and an aseptic coverslip. The prepared slide was kept in the culture dish with 20% glycerol at 25 °C, followed by microscopic observation.

Pathogenicity tests were performed as previously described [25,27]. Four wounds were uniformly distributed on the equator of each apple/pear fruit and two wounds per nectarine fruit. An aliquot of 5 μL 1 × 10^5^ conidia mL^−1^ suspension was inoculated on each wound (2 mm wide × 5 mm deep). Then the inoculated fruit were kept at 25 °C and 95% humidity for 7 d. The experiments were repeated three times with at least 6 apple/pear/nectarine fruit for each strain in each replication.

For secondary metabolism analysis, an aliquot of 1 μL conidial suspension (1 × 10^6^ conidia mL^−1^) was coated on cellophane sheets (1 × 1 cm) and cultured on PDA plates. After 1 d of incubation, the mycelia together with cellophane sheets were transferred and cultured in 24-well plates with 1 mL Czapek Yeast Extract (CY) medium per well for 2 d. The mycelia and the medium were collected for RNA extraction and HPLC assay [25], respectively. 

### 2.5. Stress Tolerance Tests

Osmotic, cell wall, membrane, and oxidative stress conditions were induced by adding 1.5 M NaCl, 6.5 mg mL^−1^ Congo red, 2 mg mL^−1^ SDS, and 2.1 mM H_2_O_2_ into the PDA, respectively. PDA buffered at pH 3.0 and pH 8.0 with 0.2 M Na_2_HPO_4_−0.1 M citric acid served as ambient pH stress conditions [25], and those buffered at pH 5.0 were used as the control. An aliquot of 5 μL 1 × 10^5^ conidia mL^−1^ suspension of each strain was incubated on PDA plates with or without stress treatments and kept for 7 d. At least three plates were used for each strain and the experiments were repeated three times.

### 2.6. Gene Relative Expression Analysis

Total RNA was extracted using TRNzol Universal Reagent (Tiangen Biotech, Beijing, China), and then reverse transcription was performed to synthesize the first-strand cDNA using a PrimeScript^TM^ RT reagent Kit with gDNA Eraser (Takara, Tokyo, Japan). Real-Time quantitative PCR (RT-qPCR) analyses were carried out with a SYBR Premix Ex Taq (Takara, Tokyo, Japan) in a Step One Plus Real-Time PCR system (Applied Biosystems, Foster City, CA, USA). The *P. expansum β-tubulin* gene was used as an endogenous control for normalization. Relative expression levels were estimated using the 2^−ΔΔCt^ method [28]. The primers used for RT-qPCR were designed with Primer Express software 3.0 and listed in Table S2. The heatmap was produced by TBtools (Toolbox for biologists) v1.0983. The experiments were repeated three times.

### 2.7. Protein Extraction and Western Blot Analysis

Mycelia were harvested after being cultured in CY medium for 24 h and ground in liquid nitrogen for protein extraction. Total protein extraction and Western blot analysis were performed as previously described [25]. Proteins were separated by 12% SDS-polyacrylamide gel electrophoresis (PAGE) electrophoresis. SYM10 antibody (Millipore, 07–412, Lake Placid, NY, USA) was used to detect sDMA and anti-β-Tubulin antibody (Abmart, M30109, Shanghai, China) served as the loading control.

### 2.8. Subcellular Localization Analysis

The generation of ∆*PeRmtC::PeRmtC-eGFP* was performed as previously described [29]. *PeRmtC* CDS without a stop codon was amplified and inserted into the eGFP vector to form a new oliC-PeRmtC-eGFP cassette. Then the recombinant vector was transformed into the ∆*PeRmtC* mutant and positive transformants were identified by PCR analysis. The primers used are listed in Table S1.

For subcellular localization observation of PeRmtC, the positive transformants were cultured and shook for 13 h. The germinated conidia were collected and stained with 4′, 6-diamidino−2-phenylindole (DAPI) (Coolaber, SL7101, Beijing, China) to locate the nucleus. A microscopic observation was carried out with a Leica laser scanning confocal microscope (Leica TCS SP5; Leica Microsystems, Wetzlar, Germany).

### 2.9. Statistical Analysis

Statistical analysis was performed using SPSS (SPSS Inc., Chicago, IL, USA). The significant differences between each strain were analyzed by a one-way analysis of variance (ANOVA) followed by Duncan’s multiple range test (*p* < 0.05).

## 3. Results

### 3.1. Identification of PRMTs Homologs in P. expansum

**Figure 1:**
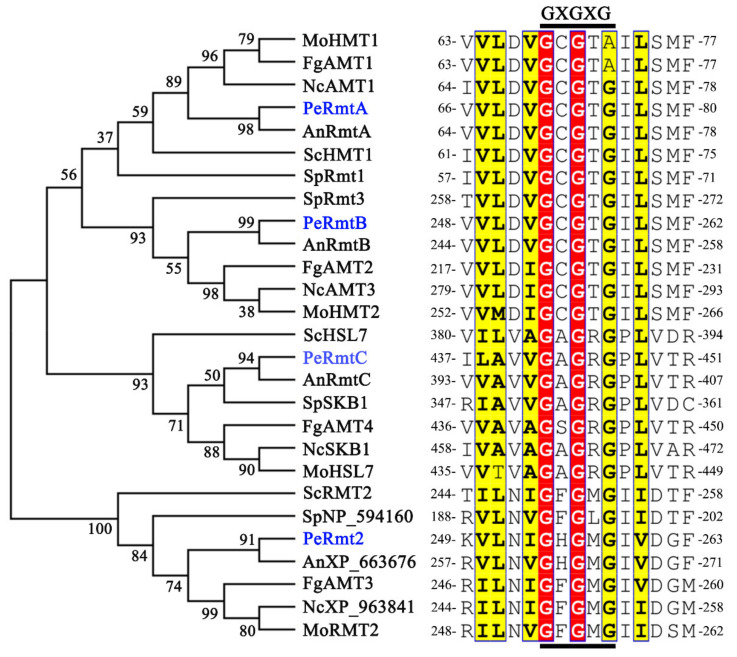
Phylogenetic analysis and conserved motif identification of PRMTs homologs in *Penicillium expansum* and six other fungal species. The phylogenetic tree was constructed by MEGA6 using a Maximum-Likelihood model. Amino acid sequence alignment was restricted to the most conserved methyltransferase motif in these fungi and performed by Clustal W. The GXGXG motif is indicated with bars. Highly conserved residues are highlighted in red and conservative residues in yellow. Pe, *P. expansum*. Sc, *Saccharomyces cerevisiae*. Sp, *Schizosaccharomyces pombe*. An, *Aspergillus nidulans*, Nc, *Neurospora crassa*. Fg, *Fusarium graminearum*. Mo, *Magnaporthe oryzae*.

Based on the amino acid sequences of ScHMT1 (CAA84976.1), SpRmt3 (NP_595572.1), ScHSL7 (CAA85090.1), and ScRMT2 (KZV12707.1) in yeast from NCBI databases, the corresponding homologs PeRmtA, PeRmtB, PeRmtC, and PeRmt2, respectively, were identified in *P. expansum*. Phylogenetic analysis was performed to compare the similarity and identity of *P. expansum* PRMTs to the respective homologs in the other six fungal species. The results revealed that all PRMTs homologs were initially divided into two clades (Figure 1). PeRmtA, PeRmtB, PeRmtC, and their corresponding PRMTs homologs were clustered together, and PeRmtA was close to PeRmtB (Figure 1). PeRmt2 and RMT2 homologs were gathered into another clade, indicating that PeRmt2 was distantly related to PeRmtA, PeRmtB, and PeRmtC. Nonetheless, all these proteins contained a highly conserved methyltransferase motif GXGXG (Figure 1) which is the core of the AdoMet-binding pocket [30].

### 3.2. PeRmtC Is Important for Conidiation and Conidial Germination 

Single deletion mutants of *PeRmtA*, *PeRmtB*, *PeRmtC*, and *PeRmt2* were constructed according to the homologous recombination method (Figure S1A). At least two deletion mutants for each of the four genes were gained and confirmed by Southern blot analysis (Figure S1B).

**Figure 2:**
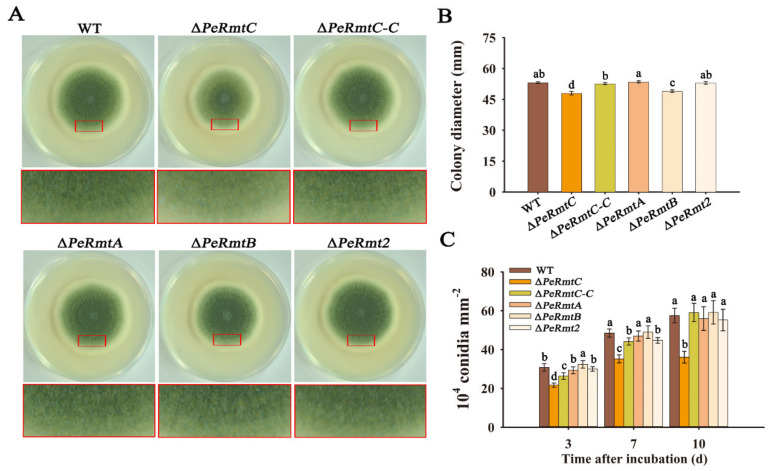
Effects of PeRmtC on growth and conidiation of *P. expansum*. (**A**) Colony morphologies of PRMTs deletion mutants on potato dextrose agar (PDA) after 7 d of incubation. Close-up views with red borders show the conidiation of corresponding strains. (**B**) Mean colony diameter of each strain after 7 d of growth. (**C**) Conidiation of each strain after 3, 7, and 10 d of incubation on PDA, respectively. The experiments were performed with three biological replicates. Columns with different letters are significantly different (*p* < 0.05).

According to Figure 2, deletion of *PeRmtC* slightly reduced mycelial growth but led to a distinct decrease in conidiation by approximately 37% after 10 d of incubation. The growth and conidiation were almost recovered to the WT level by complementing *PeRmtC* into the ∆*PeRmtC* mutant (∆*PeRmtC-C*). In addition, ∆*PeRmtB* also showed a subtle reduction in mycelial growth but no significant difference in conidial production compared with the WT strain. Deletion of *PeRmtA* or *PeRmt2* had no obvious effect on growth and development of *P. expansum*. To examine possible overlapping functions among PeRmtC and the three other PRMTs, and between PeRmtA and PeRmtB (relatively high similarity), the following double-deletion mutants were produced: ∆*PeRmtC*/∆*PeRmtA*, ∆*PeRmtC*/∆*PeRmtB*, ∆*PeRmtC*/∆*PeRmt2*, and ∆*PeRmtB*/∆*PeRmtA*. However, none of these showed a more severe impairment in conidiation than ∆*PeRmtC* (Figure S2). These results indicated that among the four PRMTs, PeRmtC played a more important role in the conidiation of *P. expansum*.

**Figure 3:**
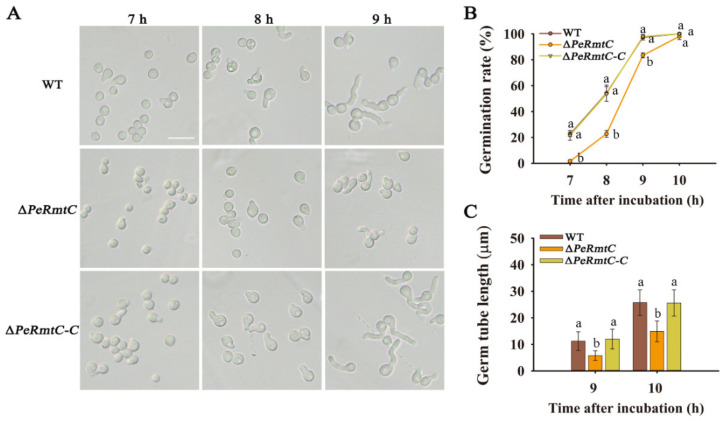
Effects of PeRmtC on conidia germination and germ tube elongation. (**A**) Morphologies of conidia germination among the WT strain, ∆*PeRmtC*, and ∆*PeRmtC-C* after 7, 8, and 9 h of incubation. The scale bar indicates 20 μm. (**B**) Conidia germination rate of each strain. (**C**) Germ tube length of each strain. The experiments were performed with three biological replicates. Values marked with different lowercase letters indicate significant differences (*p* < 0.05).

Conidial germination and germ tube growth of ∆*PeRmtC* were further evaluated (Figure 3). After 7 h of inoculation, few germinated conidia of ∆*PeRmtC* were observed; however, germination rates of the WT strain and ∆*PeRmtC-C* reached nearly 22% (Figure 3B). Additionally, the germination rate of ∆*PeRmtC* was consistently lower than that of the WT strain and ∆*PeRmtC-C* but eventually almost reached 100% after 10 h of incubation. Moreover, the germ tube length of ∆*PeRmtC* was significantly decreased compared with that of the WT strain (Figure 3C). These results indicated that deletion of *PeRmtC* delayed conidial germination and germ tube elongation.

### 3.3. PeRmtC Is Involved in Conidiophore Development 

**Figure 4:**
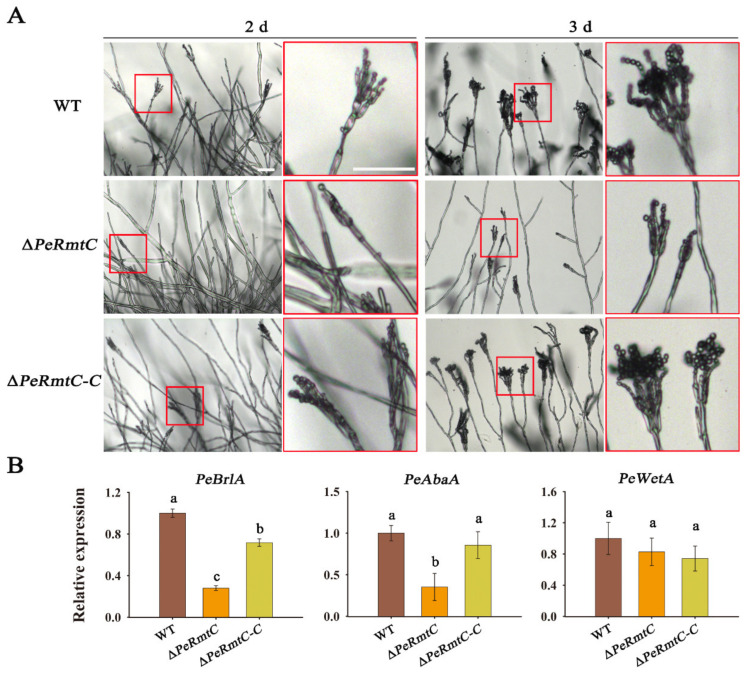
Effects of PeRmtC on conidiophore development. (**A**) Morphology observation of the conidiogenesis in the WT strain, ∆*PeRmtC*, and ∆*PeRmtC-C*. Scale bars indicate 40 μm. Close-up views with red borders show the conidiophore morphologies of corresponding strains. (**B**) Expression analysis of the core regulatory genes in conidiation. The experiments were performed with three biological replicates. Columns with different letters are significantly different (*p* < 0.05).

To further understand the role of PeRmtC in conidiation, different morphological stages of ∆*PeRmtC* were observed through a moist chamber slide culture method. After 2 d of incubation, a few conidiophores appeared in ∆*PeRmtC*, while the WT strain and ∆*PeRmtC-C* had formed a number of conidiophores with chains of conidia (Figure 4A). Chains of conidia were observed on most of the phialides in ∆*PeRmtC* after 3 d of incubation, but they were shorter than those in the WT strain and ∆*PeRmtC-C*. 

It is well known that the conserved central regulatory pathway in conidiation consists of the *brlA*→*abaA*→*wetA* cascade [31,32,33]. The relative expression levels of *PeBrlA*, *PeAbaA*, and *PeWetA* were further analyzed. *PeBrlA* and *PeAbaA* were downregulated in ∆*PeRmtC* after 1 d of incubation (Figure 4B), suggesting that *PeRmtC* may regulate conidiation of *P. expansum* via affecting the expression of *PeBrlA* and *PeAbaA* in conidiation regulatory pathway.

### 3.4. PeRmtC Affects Pathogenicity of P. expansum 

**Figure 5:**
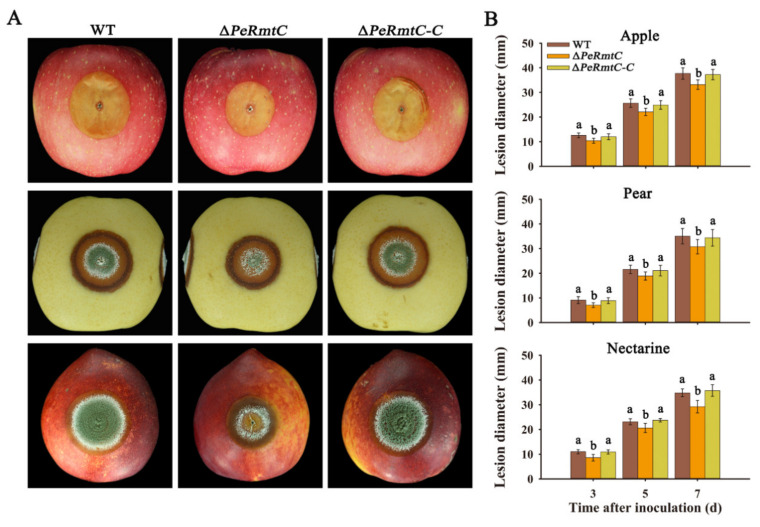
PeRmtC affects the pathogenicity of *P. expansum*. (**A**) Disease symptoms on apples, pears, and nectarines after 7 d of inoculation. (**B**) Mean lesion diameters on the fruit after 3, 5, and 7 d of inoculation. The experiments were performed with three biological replicates. Columns with different letters are significantly different (*p* < 0.05).

To explore the role of PeRmtC in the pathogenicity of *P. expansum*, ∆*PeRmtC*, the WT strain, and ∆*PeRmtC-C* were inoculated into the wounds of apples, pears, and nectarines. As shown in Figure 5, ∆*PeRmtC* exhibited 12–16% reduced lesion diameters on all fruit after 7 d of inoculation. Moreover, *PeRmtC* deletion reduced the conidiation of *P. expansum* on the surface of the necrotic lesions on pears and nectarines (Figure 5A). However, the deletion of *PeRmtA*, *PeRmtB*, or *PeRmt2* had no significant effect on the pathogenicity of *P. expansum* (Figure S3). These results indicated that PeRmtC was involved in the virulence of *P. expansum* on the host fruit. 

### 3.5. PeRmtC Plays Roles in Secondary Metabolism 

**Figure 6:**
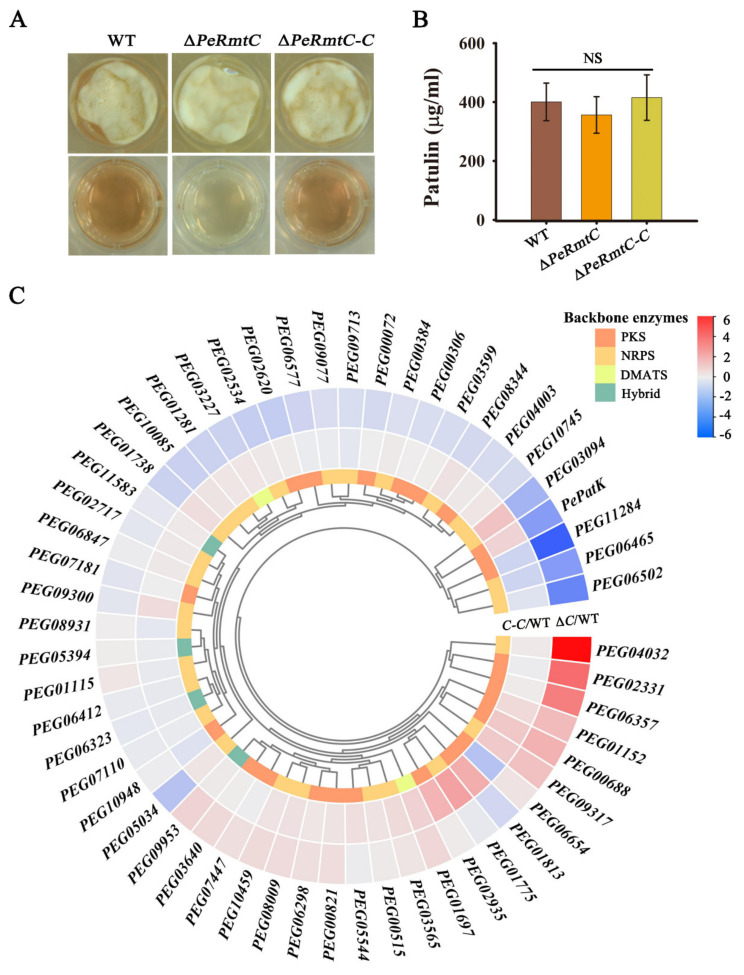
PeRmtC plays roles in secondary metabolism. (**A**) Morphologies and pigment production of the WT strain, ∆*PeRmtC*, and ∆*PeRmtC-C* after 2 d of culture on CY. (**B**) Patulin production of each strain. NS, no significant changes (*p* < 0.05). (**C**) Heatmap showing expression changes of 55 backbone genes from 55 secondary metabolite (SM) clusters. The change fold was based on the relative expression ratio as a log_2_ scale and represented by the color code. Two columns in the heatmap include *C-C*/WT (∆*PeRmtC-C* vs. WT) and ∆*C*/WT (∆*PeRmtC* vs. WT). PKS, NRPS, DMATS, and Hybrid are encoded by SM backbone genes: mean polyketide synthase, non-ribosomal peptide synthetase, dimethylallyl tryptophan synthase, and PKS-NRPS hybrid, respectively. The experiments were performed with three biological replicates.

The role of PeRmtC in secondary metabolism was determined as well. After 2 d of incubation, the color of the culture medium of ∆*PeRmtC* was lighter than that of the WT strain and ∆*PeRmtC-C* (Figure 6A). To further explore the role of PeRmtC in secondary metabolism, the expression of a total of 55 backbone genes from 55 identified secondary metabolite (SM) clusters of *P. expansum* was checked by RT-qPCR. These backbone genes were predicted to encode critical enzymes in SM biosynthesis, consisting of polyketide synthase (PKS), non-ribosomal peptide synthetase (NRPS), dimethylallyl tryptophan synthase (DMATS), and PKS-NRPS hybrid [23]. Differentially expressed genes (DEGs) were considered when the log_2_-fold change (∆*PeRmtC* or ∆*PeRmtC-C* vs. WT) was ≤ −2 or ≥ 2. Seven backbone genes were differentially expressed in ∆*PeRmtC* compared to the WT strain (Figure 6C). Four genes (*PEG11284*, *PEG06502*, *PEG06465*, and *PePatK*) were downregulated, and three (*PEG02331*, *PEG06357*, and *PEG04032*) were upregulated. Despite the downregulation of *PePatK*, which encodes the key enzyme in patulin biosynthesis, patulin production in ∆*PeRmtC* showed a subtle change without statistically significant differences compared to the WT strain (Figure 6B). Taken together, these results suggested that PeRmtC may participate in the regulation of pigment biosynthesis by regulating the expression of several SM backbone genes.

### 3.6. PeRmtC Is Involved in Stress Responses of P. expansum

**Figure 7:**
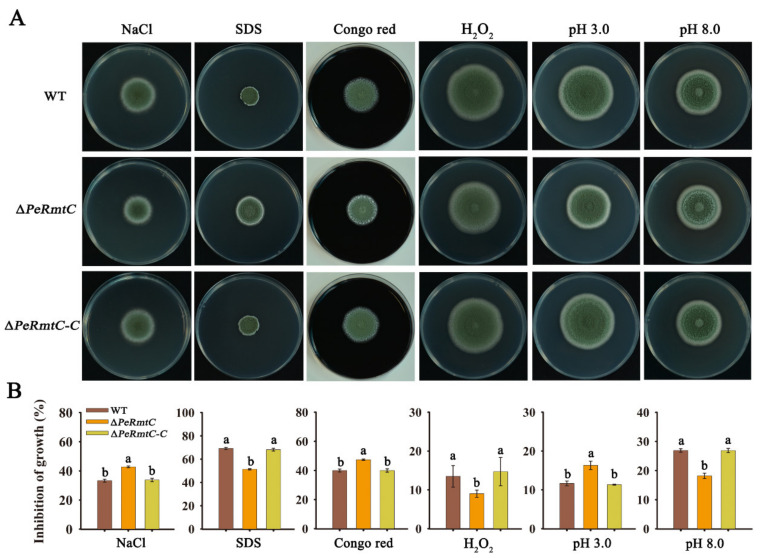
PeRmtC is involved in stress responses of *P. expansum*. (**A**) Morphologies of the WT strain, ∆*PeRmtC*, and ∆*PeRmtC-C* after 7 d of stress treatments (1.5 M NaCl for osmotic stress, 0.2 mg mL^−1^ SDS for membrane stress, 6.5 mg mL^−1^ Congo red for cell wall stress, 2.1 mM H_2_O_2_ for oxidative stress, pH 3.0 and 8.0 for ambient pH stress). (**B**) Inhibition rate of growth in different stress treatments. The experiments were performed with three biological replicates. Columns with different letters are significantly different (*p* < 0.05).

Given that fungal pathogens face various stresses during their growth and infection, tolerance tests were performed to investigate the function of PeRmtC in the stress responses of *P. expansum*. Osmotic, cell wall, membrane, oxidative, and ambient pH stress conditions were induced by NaCl, Congo red, SDS, H_2_O_2_, and Na_2_HPO_4_–citric acid, respectively. Compared with the WT strain and ∆*PeRmtC-C*, ∆*PeRmtC* exhibited increased sensitivity to NaCl, Congo red, and ambient pH 3.0 conditions, and decreased sensitivity to SDS, H_2_O_2_, and ambient pH 8.0 conditions (Figure 7), indicating that PeRmtC was associated with the cell wall and membrane integrity, and participated in responses to osmotic, oxidative and ambient pH stresses. 

### 3.7. PeRmtC Affects sDMA Levels in P. expansum

**Figure 8:**
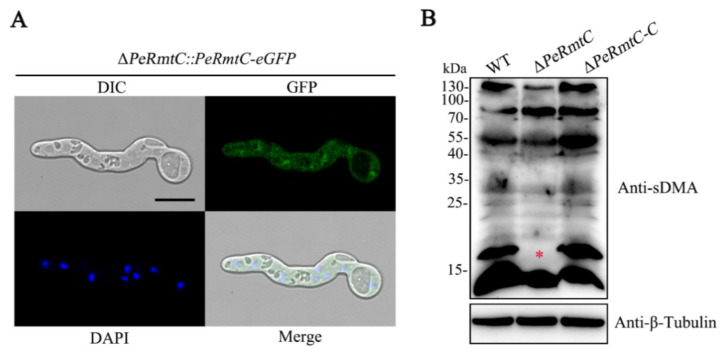
PeRmtC affects sDMA levels in *P. expansum*. (**A**) Subcellular localization of PeRmtC. DAPI, 4′, 6-diamidino−2-phenylindole. DIC, differential interference contrast. The scale bar indicates 10 μm. (**B**) Western blot analysis of symmetric ω-*N*^G^, *N’*^G^-dimethylarginine (sDMA). Total proteins were extracted and immunoblotted with SYM10 antibody for sDMA and anti-β-Tubulin antibody for the loading control. A red asterisk marks the lost methylation signal in ∆*PeRmtC*.

The C-terminal PeRmtC-eGFP fusion construct was generated and transformed into the Δ*PeRmtC* mutant to observe the subcellular localization of PeRmtC. The resulting ∆*PeRmtC::PeRmtC-eGFP* transformant had GFP signals in the cytoplasm and nucleus, as shown by the colocalization of the eGFP and DAPI fluorescence signals (Figure 8A), suggesting that PeRmtC localized to both the cytoplasm and nucleus. 

PeRmtC is homologous to mammalian PRMT5 that lays down sDMA at histones and various non-histone proteins [30]. The global levels of sDMA in the ∆*PeRmtC* were tested by the SYM10 antibody, which recognizes proteins that contain multiple symmetrically dimethylated arginines. As shown in Figure 8B, partial sDMA signals in ∆*PeRmtC* were different from those in the WT strain and ∆*PeRmtC-C*. In particular, the sDMA signal at 16–17 kDa was almost lost in ∆*PeRmtC*. This result confirms the important impact of PeRmtC on the sDMA of some proteins in *P. expansum*.

## 4. Discussion

Arginine methylation has been shown to be involved in various biological processes in fungi [15,18]. However, the roles of PRMTs in post-harvest fungal pathogens had not been previously characterized. In this study, we identified four PRMT homologs (PeRmtA, PeRmtB, PeRmtC, and PeRmt2) and proved the roles of PeRmtC in regulating the conidiation (Figure 4) and pathogenicity (Figure 5) of *P. expansum*. 

Conidia, as the primary means of thriving, can be released into the air, water, or deposited on host plants [2,33]. When conidia begin to germinate into invasive hyphae on the surface of the fruit wound, the infection process is initiated [34,35]. ∆*PeRmtC* showed a considerable reduction in conidia production and delayed conidial germination, which suggests a potential role for the PRMT5 homolog PeRmtC in regulating transmission and virulence of *P. expansum*. Nevertheless, the roles of PRMT5 homologs in other filamentous fungi are quite different except for *A. nidulans* RmtC, which can also affect conidiation and radial growth [16]. For instance, *M. oryzae* HSL7 and *F. graminearum* ATM4 had no obvious effect on vegetative growth or conidiation [18,22]. Deletion of *N. crassa skb−1* almost doubled the production of macro-conidia [21]. It is possible that evolutionary differences among fungal species led to different functions for PRMT5 homologs even though they all have the conserved arginine methyltransferase motifs.

We further found that the deletion of *PeRmtC* resulted in the late onset of conidiophore development via the downregulation of *PeBrlA* and *PeAbaA* in ∆*PeRmtC* (Figure 4). As far as is known in *Aspergillus, brlA*, *abaA*, and *wetA* are central downstream regulatory genes for conidiation [36]. The *brlA* gene, which encodes the C_2_H_2_ zinc-finger transcription factor, modulates conidiation-specific genes and initiates conidiation; *abaA* activated by BrlA is required for the differentiation and function maintenance of phialides, and *wetA* activated by AbaA is essential for spore formation and maturation [37]. A recent study demonstrated the conserved role of BrlA in the conidiophore development of *P. expansum* [38]. These findings further support that PeRmtC positively regulates conidiogenesis of *P. expansum* by mediating the two important regulatory genes *PeBrlA* and *PeAbaA*. 

Generally, secondary metabolism was interconnected with the development and associated with the pathogenicity of fungal pathogens [3]. Up to now, studies on the regulation of fungal SM biosynthesis by PRMT5 homologs are limited. Interestingly, we found that PeRmtC could affect pigment biosynthesis in *P. expansum*, whereas MoHSL7 had no effect on the colony color of *M. oryzae* [18]. Given that pigments are known to protect microorganisms against environmental stress and often have antimicrobial activity [39,40], the pigments and their roles in the growth and development *P. expansum* are worthy of identification. Our results demonstrated that PeRmtC was involved in regulating a series of SM backbone genes, most of which had never been functionally characterized in *P. expansum*. Similarly, the *A. nidulans* PRMTs disturbance led to multiple downregulated genes that are involved in secondary metabolism [15], but DEGs related to pigment biosynthesis were not mentioned. The relationship between pigment synthesis and the downregulated genes (and their gene clusters) in *P. expansum* needs to be further analyzed. 

Based on the results of subcellular localization analysis, PeRmtC was speculated to function both in the nucleus and cytoplasm, which was consistent with PRMT5 in plants and mammals [11]. PRMT5 can methylate histones to influence chromatin structure, regulate transcription, and methylate diverse non-histone proteins involved in RNA processing and transport, translation, signal transduction, DNA repair, and cellular differentiation [41,42]. We found that deletion of *PeRmtC* led to sDMA loss on 16–17 kDa proteins. However, deletion of *skb1* (PRMT5 homolog) had a minor effect on the sDMA profile in *N. crassa* [21]. Although *skb1* could cooperatively regulate sDMA with *amt1* (PRMT1 homolog), the altered sDMA profile was also different from that in ∆*PeRmtC*, suggesting that substrates of PRMT5 homologs may be specific for different fungal species. In addition, yeast Hsl7 (PRMT5 homolog) was identified to be responsible for symmetric demethylation at the histone H4 arginine 3 residue (H4R3me2s) in vivo [43]. However, we found that deletion of *PeRmtC* had no obvious impact on global H4R3me2s levels in *P. expansum* (data not shown). Identifying PeRmtC targets and exploring their contributions to biological processes in *P. expansum* will be carried out in the future. 

In conclusion, PeRmtC plays important roles in the development, pathogenicity, and secondary metabolism in *P. expansum*: it regulates conidiophore development via mediating *PeBrlA* and *PeAbaA*, affects secondary metabolism via transcriptionally controlling several SM backbone genes, and is involved in sDMA formation in *P. expansum*. Our findings are beneficial for understanding the development and pathogenesis of *P. expansum* from the perspective of arginine methylation, and broadening the exploration of targets for controlling blue mold.

## Data Availability

Not applicable.

## Supplementary Materials

The following are available online at https://www.mdpi.com/article/10.3390/jof7100807/s1, Figure S1: Construction of PRMTs deletion mutants, Figure S2: Phenotypic characterization of PRMTs double deletion mutants, Figure S3: Pathogenicity test of PRMTs deletion mutants, Table S1: The primers used for construction and identification of gene deletion, complementation, and eGFP tag strains in this study, Table S2: The primers used for RT-qPCR in this study.
