# Supplementary material for: Arginine Methyltransferase PeRmtC Regulates Development and Pathogenicity of Penicillium expansum via Mediating Key Genes in Conidiation and Secondary Metabolism
Source: J Fungi (Basel). 2021 Sep 27;7(10):807. doi: 10.3390/jof7100807 (PMC8537047; doi:10.3390/jof7100807)
Supplement: Supplementary file 1 [file jof-07-00807-s001.zip › jof-1371315-Supplementary materials.pdf]

Supplementary materials

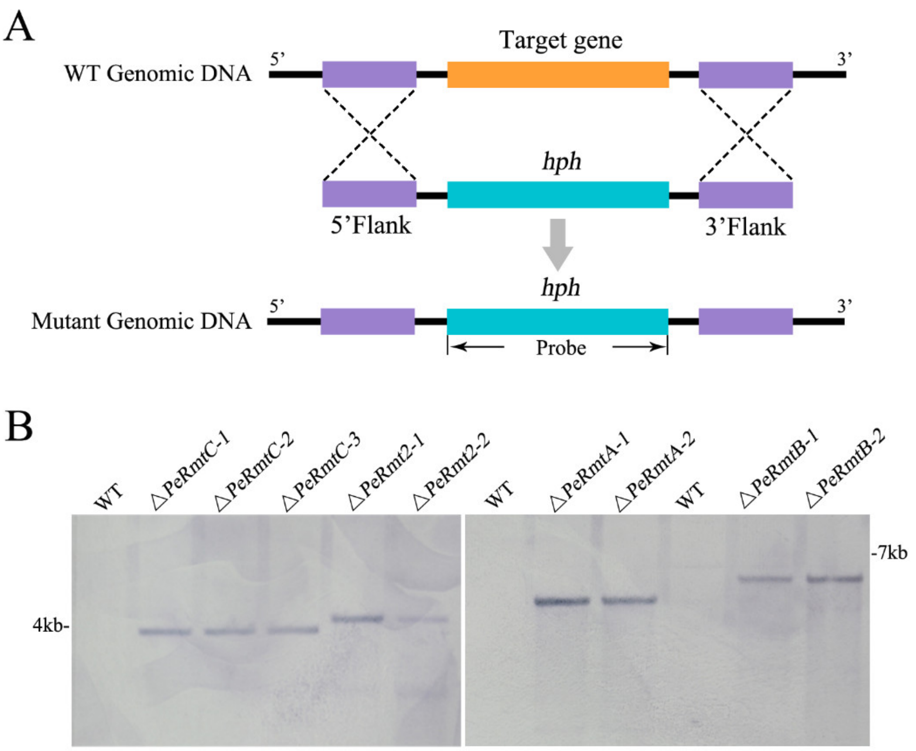

Figure S1 Construction of PRMTs deletion mutants. (**A**) Gene replacement strategy of PRMTs deletion mutants. Recombination events between the two flanking regions are indicated with crosses. (**B**) Southern blot analysis of PRMTs deletion mutants. The DNA was hybridized with an *hph*-specific probe indicated in (**A**). A single band was shown in each deletion mutant (3.96 kb, 4.23 kb, 4.89 kb, and 5.61 kb bands for Δ*PeRmtC*, Δ*PeRmt2*, Δ*PeRmtA*, and Δ*PeRmtB*, respectively) and no band in the WT strain.

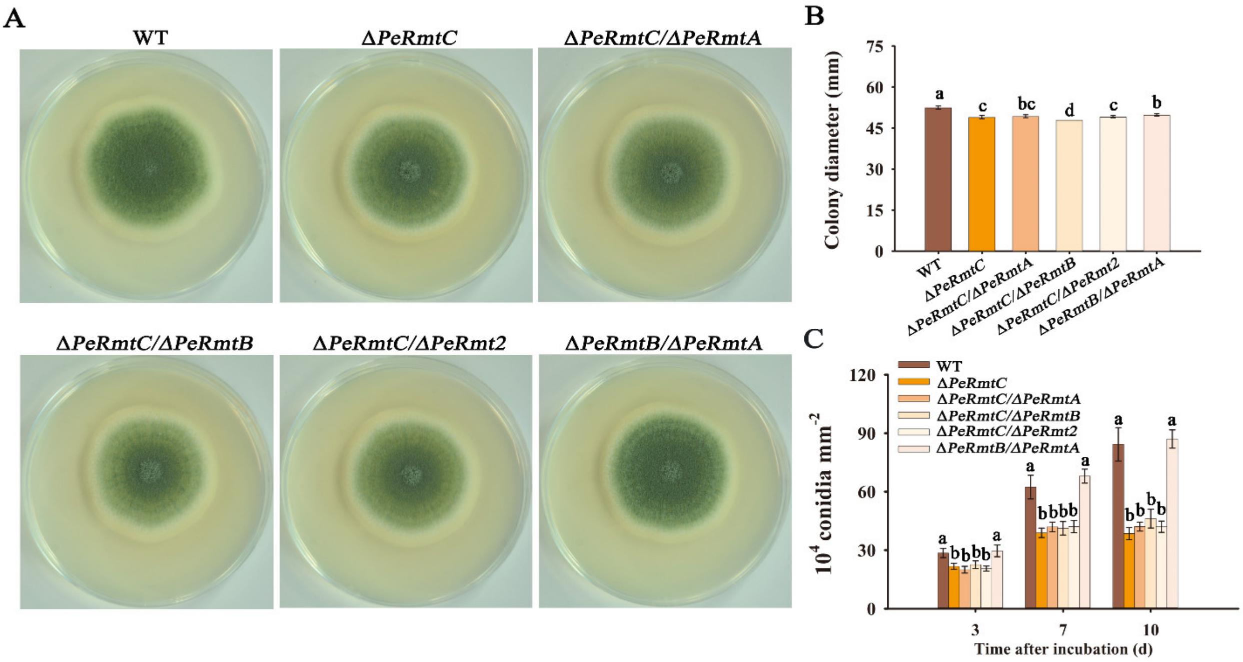

Figure S2 Phenotypic characterization of PRMTs double deletion mutants. (**A**) Colony morphologies grown on PDA after 7 d of incubation. (**B**) Mean colony diameters of the WT strain, Δ*PeRmtC*, and PRMTs double deletion mutants after 7 d of incubation. (**C**) Conidiation levels were measured by counting the number of conidia harvested after incubation for 3, 7, and 10 d. Columns with different letters are significantly different ($p < 0.05$).

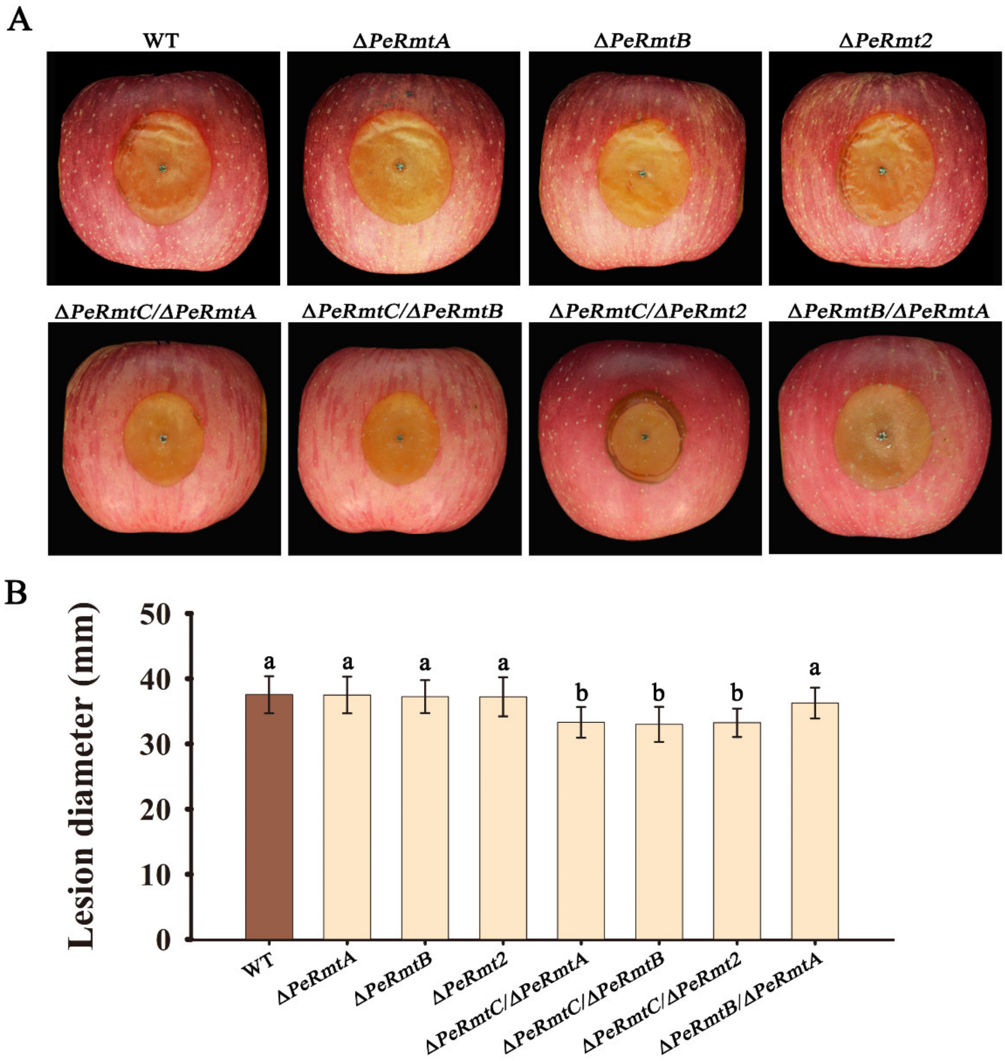

Figure S3 Pathogenicity test of PRMTs deletion mutants. Disease symptoms (**A**) and mean lesion diameters (**B**) on apple fruits after 7 d of inoculation. Columns with different letters are statistically different (p < 0.05).

Table S1 The primers used for construction and identification of gene deletion, complementation, and eGFP tag strains in this study.

Primer name	Primer sequence (5’-3’)
PeRmtA-up-F	CCAAGCTTGGAGAGCATCAAACAAATATCAGGT
PeRmtA-up-R	AGGCGCGCCTTTCGAAAGTAGTGTCAACTGATA
PeRmtA-down-F	GTCGACGCATCAAAATCGATGGTCGG
PeRmtA-down-R	CGAGCTCGCTGCTTGCTTGGCATAATCG
PeRmtA-geno-F	GCACTGTAAAAGCACACGTCC
PeRmtB-up-F	CCAAGCTTGGTACAATACAATCGCAGTGATAACAT
PeRmtB-up-R	AGGCGCGCCTGACTTATAAAGGTCAATAACAGATTG
PeRmtB-down-F	GTCGACGATCGAGCGATCTTTGGATAT
PeRmtB-down-R	GGAATTCCTGCTGGAGGATTTCGTAAGAG
PeRmtB-geno-F	CCTAATCCCTTGTCAGAGCG
PeRmtC-up-F	CAAGCTTGCAACGGACATAGCAGCCACTC
PeRmtC-up-R	AGGCGCGCCTGTTGACAGAGAAGTCCGGCG
PeRmtC-down-F	CGGATCCGAATTACCAACTAAATCTATGGCTGT
PeRmtC-down-R	CCCCGGGGTATTACCTTGTCTTGCTTGTCTTG
PeRmtC-geno-F	ATAGACCTTGATAGGACCAGCG
PeRmt2-up-F	CAAGCTTGCTCGCAGCATCCCTTCTCTTA
PeRmt2-up-R	AGGCGCGCCTGATCTGGGTGCTGTTAGTGGG
PeRmt2-down-F	CGGATCCGGGGAGATTTTGGATGATTTGAT
PeRmt2-down-R	CCCCGGGGTTTCAACATCATCACCTTCGG
PeRmt2-geno-F	GCAAGCAAAGAAACGCTGATT
Com-PeRmtC-F	GGCGCGCCCAACGGACATAGCAGCCACTC
Com-PeRmtC-R	AAGCTTTTTGTTATGCCTCCGGTCTTATG
HPH-F	TCACCCCCATCTCAACTCCA
HPH-R	TGCTCCATACAAGCCAACCAC
NEO-F	ATGATTGAACAAGATGGATTGC
NEO-R	TCAGAAGAACTCGTCAAGAAG
PeRmtC-eGFP-F	CCATCACATCACAAGAGCTCATGGAGAGCTATAATTCTTCAGAG GAT
PeRmtC-eGFP-R	TCGCCCTTGCTCACTCTAGAAAAAAAACGAACCTTCAAGGGG

Table S2 The primers used for RT-qPCR in this study.

Primer name		Primer sequence (5'-3')
PeBrlA	F	CGTGACCCCTCCTTCTTCTG
	R	TCGGTGTGGAGTAGAAGGAGTGT
PeAbaA	F	CGACCACACCCGTCACTTCT
	R	CGACAGCCGGTGAGAGCTA
PeWetA	F	CATCGCCGAATATTGCAATG
	R	GGGCTGGAGTTGGTCAAGGT
β-tubulin	F	CTCCAGCTCGAGCGTATGAAC
	R	GGCTCCAAATCGACGAGAAC
Cluster1 (PEG00072)	F	AGCCAACCGCCAATAACTTG
	R	CTCGAGGCGGGTTTGATAAA
Cluster2 (PEG00306)	F	GACCCGACAAAGAGTCCAATTC
	R	AGACTGGCGAGGCTCTGTTG
Cluster3 (PEG00384)	F	CTTCACCTCGGTGGATTTCGT
	R	AATTATAAGCATGGCCCACGTT
Cluster4 (PEG00515)	F	AAACACCGGACGCGTGATAC
	R	GGTCACGGAGGAGTACATCCA
Cluster5 (PEG00688)	F	ACTGCACTGAATGCCGTCTTT
	R	CGTTCCTTCTGTATGACCAATGTT
Cluster6 (PEG00821)	F	AAGTGGCGAGATGGAAAATCAC
	R	AGACGACCGAGATGAACACCTT
Cluster7 (PEG01115)	F	CCGGGCTCAATGAAACTTCA
	R	GTCCCTAGGCTCCGCTTGAC
Cluster8 (PEG01152)	F	GGCGCTGCAGAAATATCACA
	R	GGCAGAGCACCCCAATCAT
Cluster9 (PEG01281)	F	GTGGTTTTTGGCCACGTAGTATC
	R	AGCGGAAGGGAACTGTATTGAA
Cluster10 (PEG01697)	F	AGCTTCCCCGCGACTTCTT
	R	CCTGCCGTTCCAGCAAATAG
Cluster11 (PEG01738)	F	AAATCACGGTGGCGGATGT
	R	ATCTCGCGAACCTCTTTGCTT

Cluster12 (PEG01775)	F	CGGCAACGGGTACCTCAATA
	R	TGGCTGCTGGAGAACCTTAGA
Cluster13 (PEG01813)	F	GGGCTTCCCATCCATTTTGT
	R	TGGTTCCTAGCTGGGTAACGA
Cluster14 (PEG02331)	F	AGAGCCACGGAGCGTTCAC
	R	CTAGCGAGGCTATCGGAAAGG
Cluster15 (PEG02534)	F	ACCAGGCTCTCCCCAGTTTT
	R	CAGCTTACCAGTGGCGGTTT
Cluster16 (PEG02620)	F	ATGCGGTCCAGAGACTCGAA
	R	GTCGTTGAGAGGGATCGAGTTT
Cluster17 (PEG02717)	F	ACAACGCTGTTTTCGTGGTCTA
	R	GTGACAAGGGAGCGGTGTTC
Cluster18 (PEG02935)	F	TTGCGGGAAAACGACAAAAT
	R	TGCGCTAGGCATGGTAATACC
Cluster19 (PEG03094)	F	ACACCCACGATGCACCAAAT
	R	CAAATAAACTGGATGGCGCTTT
Cluster20 (PEG03227)	F	TTTGGTGCTCGGTGTTTGGT
	R	GCCTGTACGATGCTCCCTTTT
Cluster21 (PEG03565)	F	AGCGACGTGTCGTGGTTAGTG
	R	CTGGGCAACATCGACCTGAT
Cluster22 (PEG03599)	F	CCACTGTTCGTGCCAAAGCT
	R	TGGCTCGGACCCATAAACC
Cluster23 (PEG03640)	F	GATGCGACAGAGGCGTATCC
	R	GCCAGAACCGTCGACATTTC
Cluster24 (PePatK)	F	CCCAGGATGGCAAGACAAAC
	R	CGACTCCTGCAACGTTGAGA
Cluster25 (PEG04003)	F	CGGCGAAGCAAACTCTATCAC
	R	CCCAAGGGTTTCGCTAGTTG
Cluster26 (PEG04032)	F	CACCCAATTCCAAGGCAAAT
	R	GGCTTCGTCGGTGATATCGT

Cluster27 (PEG05034)	F	TCTGGGCAAACCTGATGTGA
	R	GACCCATGACGTTCTCCACATT
Cluster28 (PEG05394)	F	CAGTCGCTACGCCTTCAGAGT
	R	ATACCCCCATGACTTGCAGTTC
Cluster29 (PEG05544)	F	GGGTACAAATCAGGAGCACCTAGA
	R	AGAAACGCTACCCTGCACAAA
Cluster30 (PEG06298)	F	CCATCACCACCTACCCCATCT
	R	CCACGACAATCTTTCCAATATGC
Cluster31 (PEG06323)	F	CGGACGAGACAGAAGTCAAACA
	R	AACCCGCATGATCTCACCAT
Cluster32 (PEG06357)	F	AGCATGTCGTGGTTCATATGGA
	R	CAGCATGTATGACGCATTTGC
Cluster33 (PEG06412)	F	AGTTACAGCCATCCCGTAGCA
	R	AGCACGCGAGTCCACAGTCT
Cluster34 (PEG06465)	F	TCTTGGCACGCGGAATCTA
	R	ACACGGCAAGCGAGGAAATA
Cluster35 (PEG06502)	F	GAGTCAATGGGCGTTTCCAA
	R	CCATCTGACGAGCGGCTAAG
Cluster36 (PEG06577)	F	TCCCTCAAAGCCGCCTAGT
	R	CCTTCAGTTCACGCACAATCC
Cluster37 (PEG06654)	F	AGCCTCATTTCGAGCAGTTTAGAG
	R	GGGCTTCTATGCCAAGTAATGG
Cluster38 (PEG06847)	F	CAGGCGGAGGGAATGGTATA
	R	TGCGCGGTCCAGATAAAGTA
Cluster39 (PEG07110)	F	GGTCTCACAGGCGGTTAATTG
	R	TGACGGTGGGAATCACAGATT
Cluster40 (PEG07181)	F	CAAGACGCACTGGCTGCATA
	R	GCTACCCATCCCACATTGGA
Cluster41 (PEG07181)	F	ACACAGATGCAGAGACCACCAT
	R	ACGGAACTCATCCCAGATATCG

Cluster42 (PEG08009)	F	TTGCGGGTTCCGATTTGAT
	R	GTTCCTCCCCCCTTGTCTCA
Cluster43 (PEG08344)	F	ACCCACAAATACCGCGATCA
	R	CAGTAGGAAAGCGCCCAAGA
Cluster44 (PEG08931)	F	GCGTGACAACGAGGATGAGA
	R	GGCCCACTCCTGAGTCATGA
Cluster45 (PEG09077)	F	CATTTGGAGAACCACGATTCG
	R	GTCGACTTTCCAGGCGTTGT
Cluster46 (PEG09300)	F	GCAGCCAGCGTATGCACTAG
	R	TGTCTGCTCTGAACGGCAAT
Cluster47 (PEG09317)	F	GCTTGCCAATCCAGGTGTTC
	R	GCAGGAATGTCGATCGTATCAG
Cluster48 (PEG09713)	F	CCTATGGACCTGCCGAGTGTT
	R	CCGCTTTCCCAATTGTCACTT
Cluster49 (PEG09953)	F	TCATCGTCGGCCTTGGATT
	R	AGACGAGGATGTCGCAAACAG
Cluster50 (PEG10085)	F	CAACCTTCAAACCCTAGCCAAT
	R	CAGAGCATGGGAGTGGAAGAG
Cluster51 (PEG10459)	F	CCCTCAGTGGTGCCGATATT
	R	GCCGTGCACACATTCAGAGA
Cluster52 (PEG10745)	F	AAAGTTCATTCTGGCGACGATAC
	R	CCGTGTCTTTCCGGTTTGAT
Cluster53 (PEG10948)	F	TCCTAACGATATCTGGCTGCTAAA
	R	CTTTGTCACGGACCTGCAACT
Cluster54 (PEG11284)	F	GAGTGGGTGACGCCTGTGA
	R	AATGGACGTGGCAAAGCAA
Cluster55 (PEG11583)	F	ATTGATTGGGAGGCGGAAAC
	R	TGGGCTTCAAGGTGTCTTTTG
